# Sotagliflozin, the first dual SGLT inhibitor: current outlook and perspectives

**DOI:** 10.1186/s12933-019-0828-y

**Published:** 2019-02-28

**Authors:** Chiara Maria Assunta Cefalo, Francesca Cinti, Simona Moffa, Flavia Impronta, Gian Pio Sorice, Teresa Mezza, Alfredo Pontecorvi, Andrea Giaccari

**Affiliations:** 1 Center for Endocrine and Metabolic Diseases, Fondazione Policlinico Universitario Agostino Gemelli IRCCS, Rome, Italy; 2 Istituto di Patologia Speciale Medica e Semeiotica Clinica, Università Cattolica del Sacro Cuore, Rome, Italy

**Keywords:** SGLT2 inhibitors, Diabetes, Hypoglycemic therapy

## Abstract

Sotagliflozin is a dual sodium–glucose co-transporter-2 and 1 (SGLT2/1) inhibitor for the treatment of both type 1 (T1D) and type 2 diabetes (T2D). Sotagliflozin inhibits renal sodium–glucose co-transporter 2 (determining significant excretion of glucose in the urine, in the same way as other, already available SGLT-2 selective inhibitors) and intestinal SGLT-1, delaying glucose absorption and therefore reducing post prandial glucose. Well-designed clinical trials, have shown that sotagliflozin (as monotherapy or add-on therapy to other anti-hyperglycemic agents) improves glycated hemoglobin in adults with T2D, with beneficial effects on bodyweight and blood pressure. Similar results have been obtained in adults with T1D treated with either continuous subcutaneous insulin infusion or multiple daily insulin injections, even after insulin optimization. A still ongoing phase 3 study is currently evaluating the effect of sotagliflozin on cardiovascular outcomes (ClinicalTrials.gov NCT03315143). In this review we illustrate the advantages and disadvantages of dual SGLT 2/1 inhibition, in order to better characterize and investigate its mechanisms of action and potentialities.

## Introduction

Diabetes, especially type 2 diabetes, is a chronic and complex disease, which requires a multi-targeted approach tailored to the single patient. Despite the large therapeutic armamentarium available, however, this goal remains difficult to achieve.

SGLT inhibitors, a new class of oral hypoglycemic agents, have proved to be extremely effective and reliable in reducing hyperglycemia. Their insulin-independent mechanism of action inhibits sodium–glucose co-transporters, thus decreasing the renal glucose threshold (to ~ 100 mg/dL) and promoting urinary glucose excretion, without increasing the risk of hypoglycemia. Moreover, they modify several aspects of human metabolism, and some have, unexpectedly, shown great efficacy in reducing important cardiovascular events [1, 2].

The development of inhibitors targeting SGLT began with experiments with the compound phloridzin, first isolated in 1835 by French chemists from the root bark of the apple tree, and subsequently found to improve blood glucose levels in animals [3]. In 1975, DeFronzo et al. [4]. showed that phloridzin infusion in dogs increased fractional excretion of glucose by 60%, whereas glomerular filtration rate and renal plasma flow remained unchanged. Despite these results, phloridzin was abandoned as a potential treatment of type 2 diabetes due to its rapid degradation and poor absorption in the gastrointestinal tract, where it is hydrolysed into glucose and phloretin, which in turn inhibit facilitative glucose transporters, such as GLUT-1 [5], the main glucose transporter in non-insulin-dependent tissues.

SGLT is the acronym of the original name (sodium–glucose linked transporter; then modified to sodium-dependent glucose co-transporter) of a family counting at least six different isoforms in humans [6]. Of these, SGLT-1 and SGLT-2 have been widely studied because of their fundamental role in glucose and sodium transport across the brush border of gut and kidney cells, through an active mechanism exploiting the Na + electrochemical gradient generated by active sodium extrusion by the basolateral sodium/potassium-ATPase [7].

In physiologic conditions, SGLT-1 is responsible for glucose absorption in the small intestine, and for the reabsorption of nearly 10% of the filtered glucose load in the renal proximal tubule segment 3 (S3), while SGLT-2 is primarily expressed in the renal proximal tubule segment 1 and 2 (S1–S2) and is responsible for the reabsorption of ≈ 90% of the filtered glucose load [8]. Given its biological characteristics we would expect the inhibition of SGLT-2 to prevent the reabsorption of at least 80% of the glucose load. However, when SGLT2s are completely inhibited, or absent, as in knockout mice, ≈ 30–40% of glucose is still reabsorbed from urine [9]. Powell et al., observed that the absence of SGLT2s leads to a urinary glucose excretion of 30% of filtered glucose, while in SGLT2/SGLT1 double knockout mice the glycosuric effect was threefold greater [10], suggesting that when SGLT2 is inhibited, SGLT1 is forced to work at maximal transport capacity. Subsequently, Vallon’s group showed the same compensatory action of SGLT1 on renal glucose reabsorption in both acute and chronic use of SGLT2 inhibitors [11], however, robust evidence to confirm this hypothesis is still unavailable.

Several SGLT inhibitors (SGLTis) are already available in many countries (dapagliflozin, canagliflozin, empagliflozin, ipragliflozin) [12] while others are in the final phases of development (ertugliflozin, sotagliflozin). The currently available SGLT-2 inhibitors share similar pharmacokinetic characteristics and have similar effects on glycemic control; sotagliflozin, however, acts on both sodium–glucose co-transporters 1 and 2 [13]. In this review, we explore the potential advantages of sotagliflozin, compared to other selective SGLT-2is, given its unique characteristics of strong affinity for SGLT-2 and mild selectivity for SGLT-1.

## Distribution and role of SGLT-1

In a 2015 study, Vrhovac et al. [14]. used novel affinity-purified antibodies for human SGLT-1 (hSGLT-1) and SGLT-2 (hSGLT-2) to identify locations of SGLT-2 in the human kidney and SGLT-1 in the human kidney, small intestine, liver, heart and lung [8]. In the kidneys, hSGLT-2 and hSGLT-1 were found on the brush border membrane (BBM) of proximal tubule S1/S2 and S3 segments, respectively, (namely in the same location as in mice and rats). However, in contrast to rodents, hSGLT-1 was not expressed in the ascending limb of Henle and in the macula densa. Moreover, the expression of both hSGLTs was identical for both sexes. In the small intestine, hSGLT-1 was expressed on the BBM of enterocytes and subapical vesicles. Using double labeling with glucagon-like peptide 1 (GLP-1) or glucose-dependent insulinotropic peptide (GIP), SGLT-1 was also found in GLP-1-secreting L cells and GIP secreting K cells in mice. hSGLT-1 was expressed in the biliary duct cells of the liver, (as in rats). In the lungs, hSGLT-1 was found in alveolar epithelial type 2 cells and in bronchiolar Clara cells. Double labeling with aquaporin 1 immuno-localized hSGLT-1 in the human heart capillaries, instead of in the myocyte sarcolemma, as previously supposed.

These findings indicate that hSGLT-1 has several extra-renal functions, i.e. the regulation of the secretion of entero-endocrine cells, and the release of glucose from heart capillaries, which could provide cardiovascular benefits. However, the actual role played by SGLT-1 in these tissues has still not been established. Consequently, the potentially positive and negative effects of its inhibition are yet to be determined.

## Structure of sotagliflozin

**Fig. 1:**
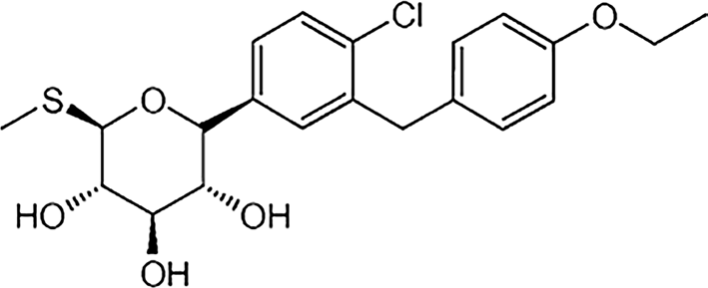
The chemical structure of sotagliflozin: (2S,3R,4R,5S,6R)-2-[4-chloro-3-[(4-ethoxyphenyl)methyl]phenyl]-6-methylsulfanyloxane-3,4,5-triol

Sotagliflozin, also known as LX4211, is a small, orally available molecule, which inhibits both SGLT-1 and SGLT-2. In humans the selectivity for SGLT-2, however, is 20-fold greater compared to SGLT-1, with an IC50 (concentration causing half of maximal inhibition) of 0.0018 µΜ and of 0.036 µM for SGLT-2 and SGLT-1, respectively [15]. Its chemical structure, (2S,3R,4R,5S,6R)-2-[4-chloro-3-[(4-ethoxyphenyl)methyl]phenyl]-6-methylsulfanyloxane-3,4,5-triol, is shown in Fig. 1.

Sotagliflozin’s effectiveness in inhibiting SGLT-2 is similar to that of the selective SGLT-2 inhibitors dapagliflozin and canagliflozin, but it is > 10-fold more potent than the latter molecules in inhibiting SGLT-1 [16]. Its effects on SGLT-1 in other tissues are, however, less known. As reported below, sotagliflozin does not seem to affect renal SGLT-1, suggesting that its low affinity has clinical effects only in tissues where SGLT-1 is highly expressed (i.e. the gut). Another possibility is that sotagliflozin acts as a potent intestinal SGLT1 inhibitor because there are higher levels of sotagliflozin in the intestinal lumen than in the general circulation.

## Myocardial SGLT-1

Evidence for the benefit of this class of drugs comes from a large real-world evidence study evaluating the risk of hospitalization for heart failure and death from any cause in patients with type 2 diabetes treated with SGLT-2 inhibitors, mainly dapagliflozin and canagliflozin [17]. Two recent clinical trials with empagliflozin and canagliflozin (EMPAREG and CANVAS, respectively), involving subjects with type 2 diabetes and high cardiovascular (CV) risk, have shown a significant reduction in the primary endpoint of Major Adverse Cardiovascular Events (MACE) and heart failure [2, 16–18]. Another clinical trial, the DECLARE-TIMI58, whose results have been recently published, showed that dapagliflozin treatment in patients with type 2 diabetes who had, or were at risk of, atherosclerotic cardiovascular disease (i.e. more than 60% of patients in primary prevention), did not show better results in preventing MACE than placebo, but did result in lower rates of cardiovascular death or hospitalization for heart failure [19]. Even though several studies are currently exploring the reasons for these interesting results [20–23]; the profound changes induced by glycosuria on substrate availability and localization in the myocardium remain unexplored [24].

Moreover, dedicated CVOTs with canagliflozin or dapagliflozin have not shown a significant reduction in all-cause mortality after treatment with these drugs [25]. Ongoing Phase III studies are exploring the cardiovascular benefits of ertugliflozin (ClinicalTrials.gov: NCT01986881) and sotagliflozin (ClinicalTrials.gov NCT03315143). In contrast to SGLT-2, SGLT-1 is reported to be highly expressed in both human autopsied hearts and murine perfused hearts (as assessed by immunostaining and immunoblotting with membrane fractionation) in the sarcolemma of cardiomyocytes [26] or more probably in the capillaries [27]. Moreover, Von Lewinski et al. demonstrated that both GLUT-4 and SGLT-1 have an insulin-dependent inotropic effects on the heart, indeed the inhibition of PI3-kinase and either SGLT1 or GLUT4 results in a loss of these functions, probably due to the reduction of glucose substrates in the cardiomyocytes.

Further, SGLT-1 and GLUT1 seem to be closely linked to each other. In protein fractions, in fact, SGLT-1 co-localizes with GLUT1, which is normally localized to the sarcolemma, and to a lesser extent with GLUT4, which can be translocated from intracellular stores to the sarcolemma when required. Despite these findings, no inhibitor effects on cardiac SGLT1, have been demonstrated, at least at therapeutic doses tested [15].

Further insight into SGLT-1’s function in the heart could derive from investigating the effects of phloridzin, a powerful dual SGLT inhibitor, unfortunately exploitable only in animal models. In recent experiments, phloridzin perfusion did not affect baseline cardiac function; however, results from its administration during ischemia–reperfusion injury suggest a possible negative effect of SGLT-1 inhibition. Indeed, the cardio-protective effect due to ischemic preconditioning was nullified when rat hearts were pre-treated with phloridzin; showing slower recovery of left ventricular contractions and a significant decrease in tissue ATP content associated with reductions in glucose uptake, as well as lactate output (indicating glycolytic flux), during ischemia–reperfusion [28]. These findings, however, were in ex vivo experiments conducted in artificial conditions, on excised murine Langendorff hearts in which the changes in overall substrate fluxes in chronic whole body SGLT-i treatments (i.e., with removal of gluco- and lipo-toxicity, increased ketones) are overwhelmed by an acute and uncompensated reduction in intracellular glucose. Nevertheless, SGLT-1 expression in the heart seems to be regulated by the overall myocardial glucose metabolism. In fact, myocardial SGLT-1 expression increases in both human subjects with end-stage cardiomyopathy secondary to type 2 diabetes and in ob/ob obese mice (a model of type 2 diabetes with altered myocardial glucose metabolism). Conversely, decreased cardiac expression of SGLT-1 was observed in STZ-treated mice, a model of type 1 diabetes (T1D). Although the reason for the divergent SGLT-1 expression in type 1 and type 2 diabetes is uncertain, it seems conceivable that increased SGLT-1 may be related to Free Fatty Acid-induced insulin-resistance, with increased SGLT-1 as an adaptation to the known reduction in cardiac GLUT1 and GLUT4 expression, resulting in hyperinsulinemia [29]. Therefore, even if sotagliflozin is able to inhibit myocardial SGLT-1, its role in restoring intracellular substrate utilization (including restored GLUT-4-mediated glucose uptake) is expected to prevail over the possible negative effect of SGLT-1 inhibition due to the reduction of glucose myocardial substrate with a consequent less viability in response to ischemia. Clearly, experiments that directly measure myocardial glucose uptake during sotagliflozin administration, and any findings on its inhibition of cardiac SGLT1 (e.g., the planned study with dapagliflozin, see Clinicaltrials.gov NCT03313752) could resolve this controversy.

A recent study to explore sotagliflozin’s effects on cardiovascular outcomes was conducted in subjects carrying a haplotype of 3 SGLT-1 loss-of-function mutations (Asn51Ser, Ala411Thr, His615Gln). The authors used a Mendelian randomization approach to estimate the 25-year effect of a reduction of 20 mg/dL in 2-h glucose through SGLT1 inhibition showing a reduction in the prevalence of obesity, and incidence of diabetes, heart failure and death [30].

Moreover, considering that recent studies have suggested that controlling postprandial glucose (PPG) could reduce the incidence of cardiovascular complications [31, 32], the improvement in PPG and glucose profile obtained with sotagliflozin may improve cardiovascular outcomes.

## Renal SGLT-1

As explained above, SGLT-2 is primarily responsible for renal glucose reabsorption (≈ 90%), while SGLT-1 accounts for the remaining 10% under physiological conditions. Given this role in renal glucose reabsorption, the expectation that SGLT-2 inhibitors would reduce renal glucose reabsorption by over 80% has been widely contradicted by clinical trials with other SGLT-2 inhibitors. Some authors have proposed SGLT-1 compensation in renal glucose reabsorption as a possible explanation [33, 34]. In fact, a double SGLT-2/SGLT-1 inhibitor like sotagliflozin would be able to overcome this problem by inhibiting glucose reabsorption more efficiently. Indeed, a study conducted by Powell’s group showed that the use of an oral SGLT-2/SGLT-1 inhibitor, known as LP-925219, was able to maximally reduce glucose renal reabsorption in SGLT-2/SGLT-1 DKO mice compared to WT, SGLT-2 KO or SGLT-1 KO mice [35].

Table 1 shows the percentage inhibition of renal glucose reabsorption at different dosages of sotagliflozin, empagliflozin and dapagliflozin, calculated from dose ranging studies in type 2 diabetic patients. Sotagliflozin seems to have a similar glycosuric effect to other selective SGLT-2 inhibitors, even if the percentage inhibition of glucose reabsorption appears numerically lower. Indeed, sotagliflozin induced UGE reaches a plateau of ~ 60 g/24 h at 200 mg daily dose, with no further increments for escalating doses (Table 1); it seems therefore conceivable that either the efficacy of sotagliflozin for renal SGLT-1 or its selectivity is too low to exert a measurable (and clinically valuable) effect.

**Table 1:** Comparison of the percentage inhibition of renal glucose reabsorption of different SGLT-inhibitors

Drug dosage	Filtered glucose (g/24 h) (eGFRx mean glucose mg/mL)	UGE (g/24 h)	Absorbed glucose (g/24 h)	Inhibition of glucose reabsorption (%)	References
Sotagliflozin 150 mg	256	36	220	14.1	Zambrowicz et al. [15]
Sotagliflozin 300 mg	287.5	47	240	16.5	
Sotagliflozin 200 mg, 200 mg bid, 400 mg	–	~60	–	–	Rosentock et al. [36]
Empagliflozin 2.5 mg	–	–	–	39	Heise et al. [37]
Empagliflozin 10 mg	–	–	–	46	
Empagliflozin 25 mg	–	–	–	58	
Empagliflozin 100 mg	–	–	–	64	
Dapagliflozin 2.5 mg	191.16	52	139.16	27.2	List et al. [38]
Dapagliflozin 5 mg	207.6	64	143.6	55.7	
Dapagliflozin 10 mg	206.1	68	138.1	54.5	
Dapagliflozin 20 mg	196.9	85	111.9	51.7	
Dapagliflozin 50 mg	191.3	82	109.3	53.1	

Inhibition of glucose reabsorption values, when not available, were calculated from the difference between filtered glucose (calculated as the product of estimated GFR [39] and estimated average glucose [40], HbA1c derived) and excreted glucose (g/24 h UGE)

Interestingly, clinical studies have shown progressive improvements in glycemic control, in terms of HbA1c, FPG and PPG, with incremental doses of sotagliflozin, in the absence of corresponding increases in UGE [36], confirming the limited action of sotagliflozin on renal SGLT-1, but also strongly suggesting that the partial intestinal SGLT-1 inhibition is clinically relevant.

A confirmation that the inhibition of intestinal (rather than renal) SGLT-1 is clinically relevant could come from testing sotagliflozin in patients with low eGFR, and therefore low filtered glucose in the kidneys. Zambrowicz et al. conducted a double-blind, placebo controlled study, to determine whether the efficacy of sotagliflozin is preserved in patients with type 2 diabetes and moderate to severe renal impairment. This study showed a significant reduction of PPG after 7 days of sotagliflozin 400 mg daily treatment, which was maintained in patients with an eGFR < 45 mL/min/1.73 m^2^ [41].

On the other hand, there was no significant improvement in HbA1c in patients with moderate renal impairment (eGFR 30–59 mL/min/1.73 m^2^) treated with dapagliflozin and canagliflozin compared to placebo [42, 43]. Similarly, empagliflozin was tested in patients with mild renal impairment (eGFR 30–60 mL/min/1.73 m^2^) with only minor results [44]. It is thus clear that the dependence of selective SGLT-2 inhibitors on renal function represents a limitation in the efficacy of these drugs in patients with renal impairment. Considering that type 2 diabetes is a major risk factor for chronic kidney disease and that glycemic control can reduce the risk of microvascular complications and the progression of chronic kidney disease (CKD) [45], the use of sotagliflozin in patients with CKD represents an important new therapeutic opportunity.

## Intestinal SGLT-1

By inhibiting SGLT-1, sotagliflozin delays glucose absorption in the distal small intestine and colon, consequently reducing Post Prandial Glucose (PPG) and improving glycemic control in type 2 diabetes subjects [15, 41].

Treatment with increasing doses of canagliflozin (an SGLT-2 inhibitor with low potency SGLT-1 inhibition) given 10 min before a mixed meal, produced a significant reduction in PPG only with doses higher than 200 mg; this effect, however, was observed only for the first meal after dosing [9]. Therefore, a subsequent study, conducted to investigate the effect of a single 300 mg dose of canagliflozin on intestinal glucose absorption, demonstrated that this dosage reduces PPG and insulin concentration in healthy subjects, delaying the rate of appearance of oral glucose, likely through the transient inhibition of intestinal SGLT-1 [46].

None of the studies published so far has identified the real mechanism by which sotagliflozin inhibits SGLT-1. Importantly, it is unclear whether sotagliflozin interacts with SGLT-1 only locally (interacting with the co-transporters on the extracellular intestinal luminal side), as reported by Wright et al. [47] or also systemically, after intestinal absorption of the drug. In the first case, the effects of sotagliflozin on PPG should be evident a few hours after drug ingestion, independently of its plasma half-life. Daily drug administration, however, has shown positive effects on both fasting plasma glucose (FPG) and PPG.

In a dose-ranging study, the efficacy, safety and tolerability of four oral doses of sotagliflozin (75 mg once daily, 200 mg once daily, and 200 mg twice daily and 400 mg once daily) were tested in patients with type 2 diabetes inadequately controlled on metformin. Sotagliflozin improved glycemic control by reducing HbA1c and FPG in a dose dependent manner throughout the 12-week observation period (maximum efficacy was achieved with 400 mg once daily), with no corresponding increase in urinary glucose excretion (UGE) suggesting that the greater efficacy observed with 400 mg once daily dose compared to 200 mg daily dose, is achieved through clinically meaningful SGLT1 inhibition in the GI tract [36].

In a previous study [15] the authors showed that treatment with LX4211 (150 mg or 300 mg daily) also significantly lowered 2-h postprandial glucose (PPG) levels in each treatment group as compared with placebo with no difference between the used doses; moreover an increase in GLP-1 and PYY has been observed from 0 to 13 h after meal in subjects treated with 300 mg single dose LX4211 suggesting (but not demonstrating) that the post-prandial effect might also be obtained with q.d. dosing.

In an explorative study, Brian Zambrowicz et al. [48] examined the efficacy of sotagliflozin (alone or in combination with sitagliptin) in both mice and humans. Results from the study seem to suggest that the inhibition of SGLT-1 by sotagliflozin lasts longer than the first meal.

**Fig. 2:**
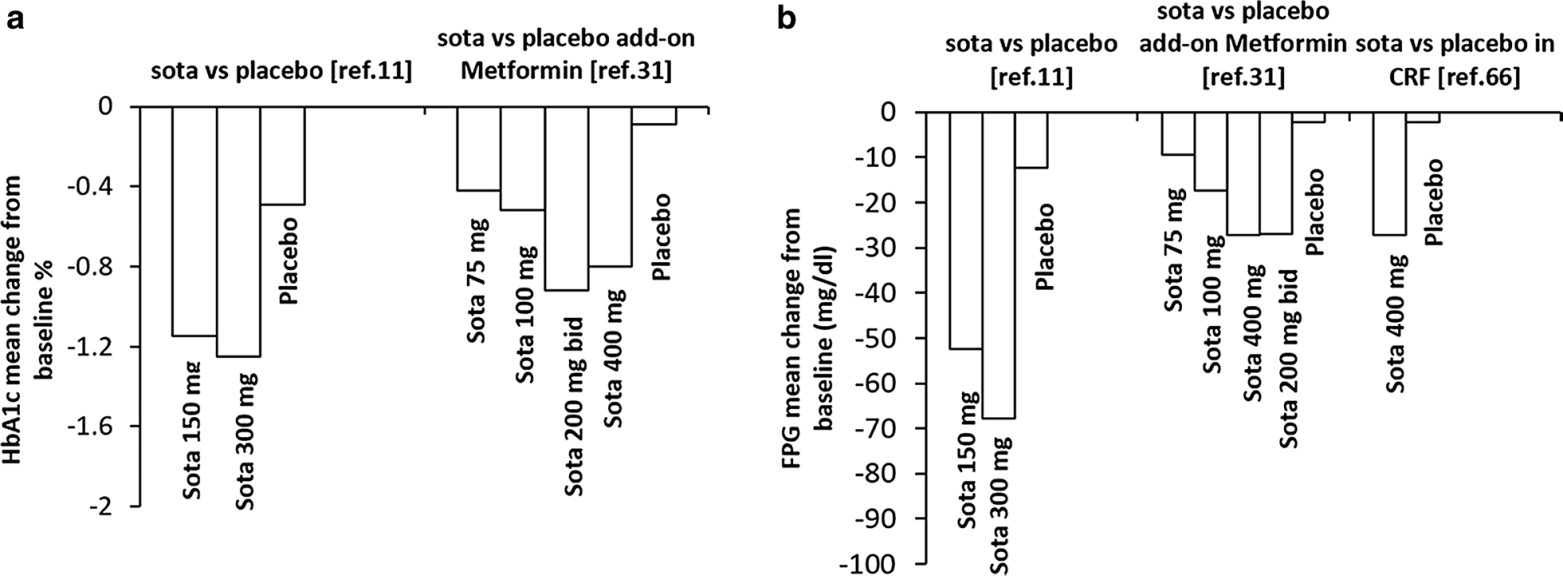
Comparison of the effect of sotagliflozin on HbA1c (**a**) and fasting plasma glucose (**b**) in various clinical trials on type 2 diabetic patients

The effect of sotagliflozin on main outcomes in type 2 diabetes subjects, as reported in the main clinical trials available, is shown in Fig. 2.

The hypothesized mechanism is that sotagliflozin’s partial inhibition of intestinal SGLT1 results in delayed glucose uptake, which induces the release of GLP-1 and PYY by L cells, and that the increased levels of active GLP-1 may contribute to improved glycemic control in treated patients [49]. However, a double tracer study to measure glucose transit and absorption throughout the day, with (various) sotagliflozin dosages, is probably needed, at least to demonstrate its important intestinal effects.

Initial studies in mice treated with LX 2761, a non-absorbable SGLT-1 inhibitor, showed reductions in fasting plasma glucose (FPG) and PPG, improvement in glycemic control, without increased glucosuria, and an increase in circulating levels of GLP-1 and peptide YY (PYY), hormones that suppress the appetite [50].

In a rodent model, Hara et al. demonstrated that oral administration of canagliflozin suppresses GIP secretion, by inhibiting SGLT-1 in the upper part of the intestine, and reduces the excursion of blood glucose under hyperglycemic conditions (OGTT), by increasing total GLP-1 levels. Interestingly, the action of canagliflozin on GLP-1-producing cells may be determined by increased glucose delivery to the lower part of the small intestine [51]. Since this increase in GLP-1 is not observed with more SGLT-2 selective inhibitors, such as empagliflozin [52], it is safe to assume that the increase in GLP-1 is a consequence of intestinal SGLT-1 and subsequent delayed glucose absorption.

Additionally, it is well established that delaying glucose absorption in more distal parts of the gut increases GLP-1 secretion [53]. In a single-dose study with sotagliflozin at 300 mg in 12 patients with type 2 diabetes, there was a significant increase in GLP-1 and PYY, accompanied by a significant decrease in plasma glucose and insulin doses [15]. Nevertheless, the sotagliflozin-induced increase in GLP-1 levels following a glucose load in mice treated with this compound and in SGLT-1 knockout (−/−) but not in SGLT-2 knockout (−/−) mice strongly suggests, at least in a murine model, that SGLT-1 inhibition elicits GLP-1 secretion from intestinal L cells [54] leading to increased glucose and decreased pH in cecal (large colon) contents. Further, delayed glucose absorption in the gut might make more glucose available to intestinal microbiota. Glucose fermentation in the colon produces short-chain fatty acids (SCFAs), a potential trigger for GLP-1 secretion [55, 56]. Previous studies have shown that ingestion of dietary-resistant starch, which delays glucose absorption, leads to an increased production of SCFAs, through glucose fermentation, and enhances circulating levels of GLP-1 and PYY in rodents [57]. Interestingly, sotagliflozin-treated mice (and SGLT-1 ko mice) have a lower pH of cecal contents after glucose challenge, suggesting that sotagliflozin reduces plasma glucose by triggering GLP-1 secretion through intestinal SCFA production [50].

GLP-1 secretion from L cells, however, is not solely due to the presence of glucose in the intestinal lumen and direct contact between the substrate and secreting cells. As an example, GLP-1 is secreted in a biphasic pattern: the first peak appears 15–30 min after the meal (earlier than the appearance of glucose in the lower gut), and is followed by second peak at 90–120 min [58]. Moreover, murine data show an involvement of cholinergic neurons in the upper intestine, which may stimulate GLP-1 secretion in L cells in the lower intestine [59]. Other studies have suggested that SGLT3, a glucose sensor that does not transport glucose, expressed in the upper small intestine may modulate the afferent vagal nerve to finally stimulate GLP-1 release [60]. Whatever the mechanism(s), it appears evident that at least part of sotagliflozin’s effect on glucose metabolism could be mediated by an increase in GLP-1 secretion, although the actual contribution needs to be experimentally measured.

Finally, a blood intestine recirculation through bile (prolonging intestinal interaction between sotagliflozin and SGLT-1) might be hypothesized.

This scenario is, however, challenged by a recent study by Solini et al. [61]. In their in vitro model, the authors showed that dapagliflozin (a highly selective SGLT-2 inhibitor) increases glucagon by acutely upregulating SGLT-1 expression in islet alpha cells independently of SGLT-2 inhibition.

Acarbose stimulates GLP-1 secretion in both healthy and diabetic subjects [62]. Therefore, sotagliflozin seems, at least in part, to share the effects of acarbose.

**Fig. 3:**
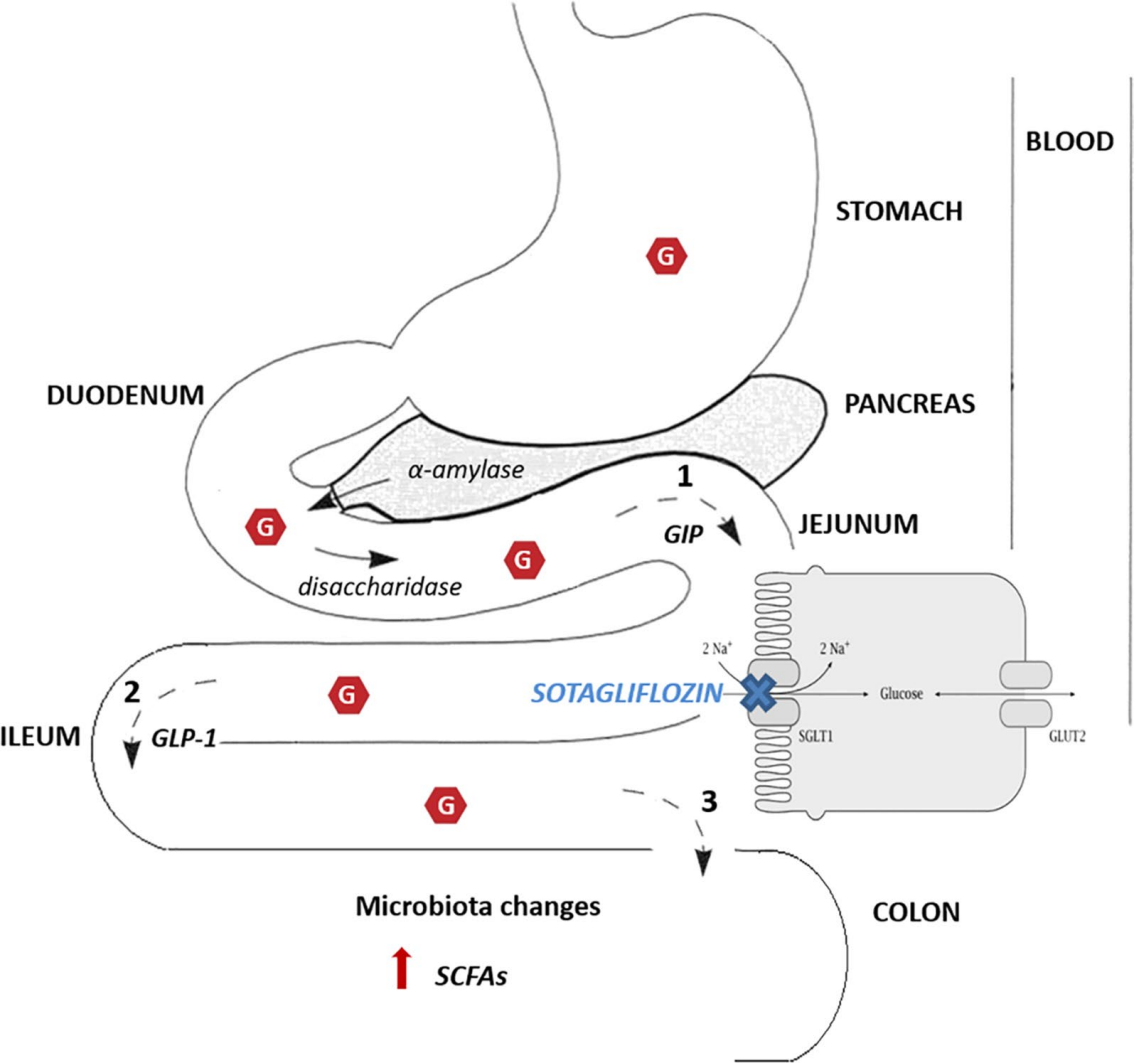
Intestinal effects of SGLT-1 inhibition by sotagliflozin. By inhibiting SGLT-1 sotagliflozin reduces PPG and improves glycemic control. Possible mechanisms are: (1) delayed glucose absorption in the distal small intestine; (2) consequent increased GLP-1 secretion by L cells, mostly located in the cecum, and (3) delayed glucose in the colon where it could promote changes in microbiota and increase production of SCFAs; the latter seems to independently increase GLP-1 secretion. *G* glucose, *GIP* gastric inhibitory peptide, *GLP* glucagon like peptide, *SCFAs* short chain fatty acids

Metformin could represent another possible factor interfering with glucose handling in the gut [63]. Recent human data have shown that metformin modifies bile acid recirculation and gut microbiota resulting in enhanced entero-endocrine hormone secretion [64]. Moreover, the higher potency of Delayed-Release (DR) metformin formulations (characterized by higher metformin availability in the distal gut, with less systemic absorption [65]) clearly indicate the predominant lower bowel-mediated mechanism of action of this widely-used drug, eventually stimulating GLP-1 secretion [66]. Further, metformin seems to have a direct action on microbiota, determining a significant increase of protective bacterial genera such as *Akkermansia muciniphila* and *Clostridium cocleatum* in mice. In humans with type 2 diabetes, metformin treatment promotes the restoration of relative abundance of specific genera, such as Akkermansia and Lactobacillus [67, 68]. It appears obvious that delayed glucose absorption in the lower gut (sotagliflozin) with a concomitant change in lower gut microbiota (metformin) could reciprocally interact. Whether this interaction really modulates glucose metabolism is still unknown. A schematic overview of intestinal sotagliflozin effects is shown below (Fig. 3).

## Dual SGLT-1 and DPP-4 inhibition

Given that SGLT-1 inhibition enhances GLP-1 secretion and DPP-4 inhibition prolongs endogenous GLP-1 half-life, a synergic effect on glucose control in type 2 diabetes is to be expected. In patients with type 2 diabetes, administration of canagliflozin (100 mg daily for 3 days) led to a twofold increase in GLP1 levels from baseline, and concomitant treatment with tenelegliptin led to a fourfold increase in GLP-1 levels of from baseline [69, 70]. Intriguingly, an improvement in beta cell incretin sensitivity has been described in Type 2 diabetes patients treated with dapagliflozin [71], and a mild increase in GLP-1 levels has also been observed with empagliflozin. These gliflozins, however, have no inhibitory action on SGLT-1 [72, 73] and should not therefore be able to directly increase GLP-1 secretion. Recent data from Bonner et al. demonstrated that SGLT-1 and SGLT-2 are specifically expressed in pancreatic alpha cells [74], where SGLT-2 inhibition determines increased glucagon secretion. The recent discovery that pancreatic alpha cells secrete GLP-1 [75], with possible prevailing paracrine effects, makes this mechanism particularly interesting.

The utility of co-administering DPP-4 inhibitors and SGLT-2 inhibitors is now well established [76–78], although with apparently less than additive efficacy. As mentioned above, sotagliflozin enhances GLP-1 secretion, and a higher efficacy is therefore expected when combined with a DPP-4 inhibitor. In a first, explorative study, Zambrowicz et al. [48] confirmed these expectations both in mice and in humans. Obese C57BL6J mice were treated with sotagliflozin 60 mg/kg, sitagliptin 30 mg/Kg, a combination of both or inactive vehicle, and an open-label, 3 treatment, 3 crossover study was conducted at a single center, where patients with type 2 diabetes randomly received sotagliflozin, sitagliptin or a combination of both (ClinicalTrials.gov identifier: NCT01441232). A significant increase in active GLP-1, after a meal challenge containing glucose, was observed in the combination therapy groups compared to the others, suggesting a synergic effect of the two drugs. Unfortunately, the study was too short (2 weeks) to demonstrate the synergic effect of the two inhibitions on HbA1c levels. To date, another clinical trial, exploring the addition of sotagliflozin (as compared with empagliflozin) in patients taking a DPP-4 inhibitor alone or with metformin (SOTA-EMPA) is currently recruiting (ClinicalTrials.gov identifier: NCT03351478.

Moreover, an adjuvant and additional effect on the glycaemic control and body weight of a combination therapy with SGLT-2 and GLP1-RA is also desirable as reported in recent clinical trial [79, 80]. Future studies investigating the effects of combination therapy with GLP-1 and sotagliflozin might give positive and stronger results.

## The use of sotagliflozin in type 1 diabetes

Despite recent advances in the treatment of type 1 diabetes due to the introduction of new fast-acting and basal insulin analogs, insulin pumps and the possibility of continuous glucose monitoring (CGM) the majority of type 1 diabetic patients do not achieve and maintain adequate glycosylated hemoglobin levels [81]. While almost all new drugs for type 2 diabetes lose their efficacy in presence of euglycemia, thus reducing the risk of hypoglycemia, the latter still represents one of the major barriers to achieving euglycemia in type 1 diabetes. Randomized controlled trials assessing the adjunctive use of liraglutide, sitagliptin and metformin in type 1 diabetes patients have not shown consistent or sustained reductions in glycated hemoglobin levels or increases in episodes of severe hypoglycemia or diabetic ketoacidosis [82–84]. Since the blood glucose lowering effect of SGLT inhibition is minimal in the presence of euglycemia, and since the euglycemic action of these drugs is completely insulin independent, SGLT inhibitors might theoretically represent a valid adjunctive therapy in patients with type 1 diabetes.

In a type 1 diabetes rat model [85], SGLT-2 inhibitors were reported to improve blood glucose levels, increase UGE and protect remaining islet β-cell function by reducing the oxidative stress induced by glucose toxicity. Several studies with selective SGLT-2 inhibitors have been conducted in patients with Type 1 diabetes, confirming the efficacy of this new approach [86–88].

The effect of sotagliflozin on glycemic control was first evaluated in a type 1 diabetes mouse model [89]. In this study, sotagliflozin notably improved glycemic control in diabetic mice, maintained on a low insulin dose, without increasing hypoglycemic events. Subsequently, several studies were conducted to assess the effects of sotagliflozin added to insulin therapy in subjects with type 1 diabetes.

**Fig. 4:**
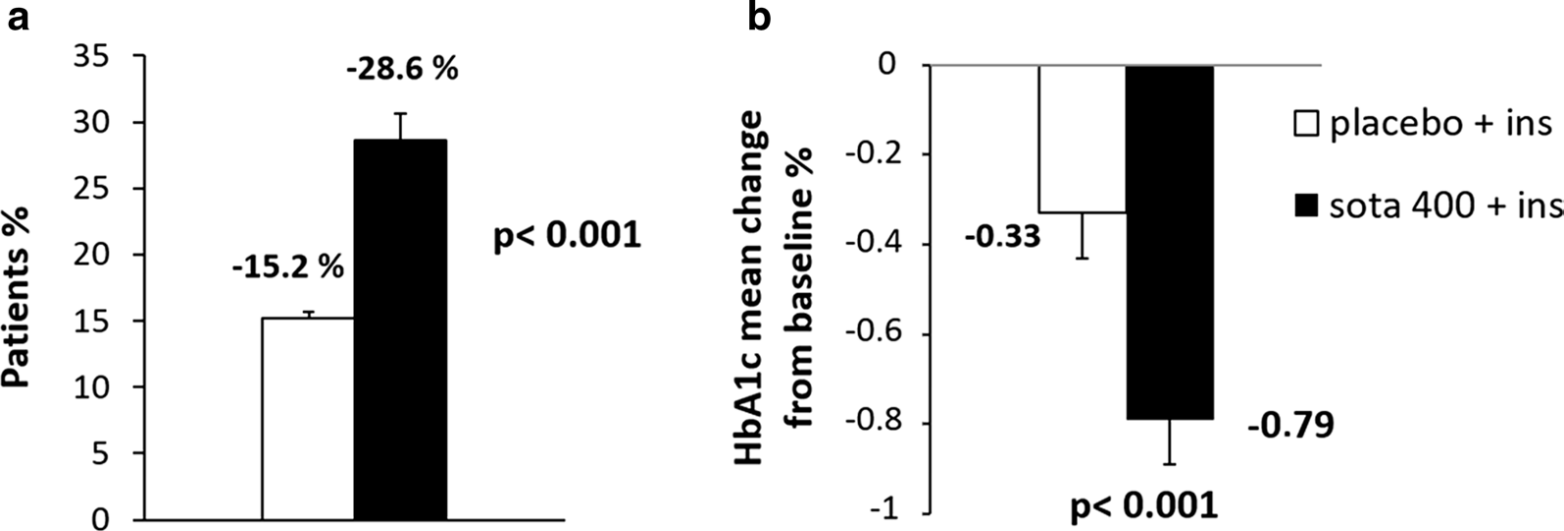
Percent of patients reaching the primary end point (**a**) and reduction in glycated hemoglobin level (**b**) after 24 weeks of intervention in the sotagliflozin group compared to placebo group as reported in inTandem 3 trial

In the main trial, inTandem 3 [90] the primary end point was a glycated hemoglobin level lower than 7.0% at week 24, without episodes of severe hypoglycemia or diabetic ketoacidosis, while secondary end points were the change from baseline to week 24 in glycated hemoglobin level and the possible reduction in daily bolus insulin dose, body weight and systolic blood pressure. Up to 28.6% of the sotagliflozin group achieved the challenging primary end point (HbA1c < 7% at week 24, with no episodes of severe hypoglycemia or diabetic ketoacidosis), with a greater reduction in the glycated hemoglobin level from baseline in the sotagliflozin group than in the placebo group (difference, − 0.46 percentage points; P < 0.001) (Fig. 4). In the sotagliflozin group, the placebo-corrected reductions from baseline in the mean daily total, bolus, and basal doses of insulin were − 5.3 units per day (− 9.7%), − 2.8 units per day (− 12.3%), and − 2.6 units per day (− 9.9%), respectively (P < 0.001 for all comparisons). The decrease in body weight and in systolic blood pressure from baseline to week 24 was significantly higher in the sotagliflozin group than in the placebo group (difference, − 2.98 kg; P < 0.001; difference, − 3.5 mmHg; P = 0.002 respectively).

**Fig. 5:**
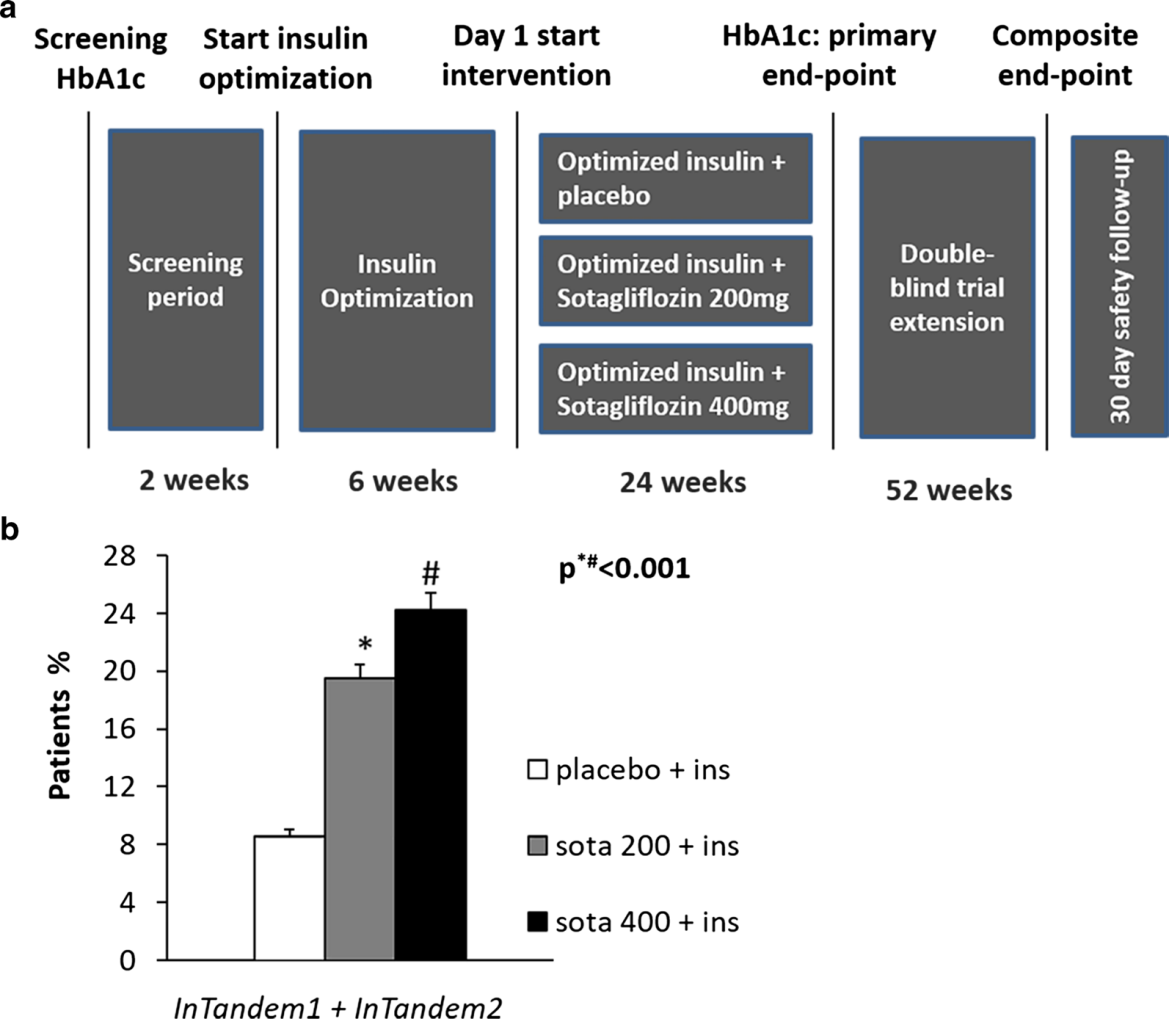
inTandem 1 and inTandem 2 clinical trials: study design (**a**) and percentage of patients that achieve composite endpoints (Hba1c < 7%, no weight gain, no severe hypoglycemic event or diabetic ketoacidosis) at week 52 (**b**). P^#^ = sota 200 mg vs placebo; P* = sota 400 mg vs placebo

Sands et al. conducted another randomized, multicenter, placebo-controlled, double-blind study to evaluate sotagliflozin, as adjunct therapy in adult subjects with type 1 diabetes [91]. The primary outcome of the study was the effect of sotagliflozin therapy on change from baseline of total daily bolus insulin dose during the treatment period. The results demonstrated a percent variation from baseline in total daily bolus insulin administration that was − 32.0% in the sotagliflozin group and − 6.4% in the placebo group (P = 0.007) respectively. HbA1c decreased by 0.55% from baseline after treatment with sotagliflozin, compared with 0.06% in the placebo group (P = 0.002). Moreover, sotagliflozin treatment also reduced mean body weight compared with an increase in the placebo group (− 1.7 kg vs 0.5 kg) (P = 0.005). The results of other two Phase 3 clinical trials in the sotagliflozin T1D program, inTandem1 (ClinicalTrials.gov: NCT02384941) and inTandem2 (ClinicalTrials.gov: NCT02421510), have been recently published. Both trials aim to evaluate efficacy (change from baseline to week 24 in glycated hemoglobin level, daily bolus insulin, fasting plasma glucose, body weight) and safety (number of severe hypoglycemia [1] and diabetic ketoacidosis [DKA] episodes) of the use of sotagliflozin, 200 mg or 400 mg, in adult patients with type 1 diabetes. The novelty of these studies, compared to inTandem3, is to provide, as shown in the study design in Fig. 5, a preliminary 6-week phase for the optimization of insulin therapy.

In summary, treatment with sotagliflozin in combination with insulin in subjects with type 1 diabetes, significantly decreased glycated hemoglobin level, insulin dosage, body weight and systolic blood pressure, and, more importantly, without increasing the occurrence of hypoglycemia. Its key effect could be related to the reduction in postprandial glucose, which consequently leads patients on sotagliflozin to decrease insulin bolus.

## Diabetic ketoacidosis and other side effects

As with other SGLT-2 inhibitors [92], the use of sotagliflozin leads to an increase in side effects triggered by glycosuria, e.g. genital mycotic infections, (the most common issue). Moreover, consistent with its inhibitory effect on SGLT-1 receptors, sotagliflozin is also accompanied by gastrointestinal side effects as reported in the inTandem 3 trial [90] (diarrhea occurred in 4.1% of the sotagliflozin group vs 2.3% in the placebo group). Besides these two rare and well tolerated side effects, various clinical trials have raised another important concern: the risk of hypoglycemia and ketoacidosis (DKA). In the cited phase 3, double-blind trial, whose results were published by Garg et al. [90], 1402 type 1 diabetes patients were randomly assigned to received insulin therapy or sotagliflozin (400 mg daily) or placebo for 24 weeks, reporting a lower event rate of documented hypoglycemia with a blood glucose level of less than 55 mg per deciliter or lower in sotagliflozin group compared to placebo, while the percentage of patients who had an episode of documented hypoglycemia with a blood glucose level of 70 mg per deciliter or lower was similar in the sotagliflozin group and the placebo group (96.3% [673 of 699 patients] and 95.3% [670 of 703], respectively). Similar results were obtained in a study conducted by Pier et al., in which the efficacy and safety of empagliflozin therapy (2.5 mg; 10 mg and 25 mg) compared to placebo was evaluated in type 1 diabetes patients. The rate of symptomatic hypoglycaemic episodes with glucose ≤ 3.0 mmol/L, not requiring assis-tance, was similar between groups over 30 days [93]. Similarly, treatment with dapagliflozin determined a comparable incidence of hypoglycaemia events in patients treated with different doses of SGLT2-inhibitor compared to placebo (0.1% in each of the dapagliflozin groups vs 0.1%). Moreover, most hypoglycemic events were from add-on to insulin and add-on to sulfonylureas therapies [94].

Moreover in the inTandem 3 study, the sotagliflozin group had a higher incidence of ketoacidosis episodes compared to the placebo group (3.0% vs 0.6%). In fact, 1.6% of subjects in the sotagliflozin group discontinued the trial due to DKA vs 0.1% of the placebo group. Although the definition of DKA differed between trials, similar occurrence of DKA episodes have been described with the use of dapagliflozin [95].

A formal warning for the increased risk of DKA with SGLTs has been issued by the FDA [96]. Different mechanisms have been proposed to explain this important side effect. First, the reduced glucose concentration partially switches ATP generation from glucose to FFA (Free fatty acids); while this mechanism is responsible for loss of fat mass, it might also determine a mild increase in the genesis of ketones (similar to that usually reached during fasting). Further, the reduced insulin dose, when SGLT-2 inhibitors are associated with insulin therapy, may not be sufficient to block gluconeogenesis in the liver, with a net consumption of oxaloacetate (for gluconeogenesis) impeding the entrance of FFA-derived AcCoA, which in turn are converted to ketones. A vicious circle may also be induced by the inhibition of SGLT-2 in alpha cells which leads to an increase in glucagon with consequent stimulation of ketogenesis in the liver [61]. Moreover, a reduction in renal clearance of ketones with SGLT-2 inhibition has been described in animal models [97] and may represent another mechanism responsible for DKA with SGLT-2 inhibitor drugs.

A recently published meta-analysis of the three hallmark CV outcome trials (EMPA-REG OUTCOME, CANVAS, DECLARE TIMI-58) has documented, despite the unquestionable benefits on reducing hospitalisation for heart failure and progression of renal disease, a two-fold increase in the risk of DKA with the use of this drug class [98]. However, expecially in type 1 diabetes, instead of considering SGLT inhibitors as responsible for the increase in DKA episodes, patients treated with SGLTis should be considered as more vulnerable to DKA episodes. Indeed, ketoacidosis per se represents a known risk in type 1 diabetes. A recent systematic review of the literature, found a 5 to 10% overall prevalence of ketoacidosis in type 1 diabetic adult patients [99], comparable to the incidence of DKA observed in the SGLT inhibitor trials [100–102]. Monitoring for ketosis, discontinuation of SGLTis before surgical procedures, and caution during stressful situations could be sufficient to mitigate the risk of DKA during SGLTi treatment, especially for patients on insulin pump therapy, in which a malfunction of the infusion set may increase the risk of DKA. With proper education, these episodes are probably entirely avoidable. And the benefits obtained with SGLTi treatment certainly outweigh the risks, especially if the latter are well understood and managed by both physicians and patients. Further, by delaying glucose absorption, sotagliflozin may reduce the need for ultra-fast insulin [103] in type 1 diabetes.

## Conclusions

Although the specific CardioVascular Outcome Trial is still under way, it can be affirmed that sotagliflozin seems to share all the advantages of the other, already available, SGLT-2 selective inhibitors. In contrast with other SGLT-2is, however, sotagliflozin has the added advantage of delaying glucose absorption in the intestine. Although it is evident that this inhibition may be important in reducing post-prandial glucose (or at least in delaying and lowering the glucose peak), too many pathophysiologic mechanisms are still poorly studied. More importantly, the possible interactions of sotagliflozin with other common drugs (metformin, interacting through microbiota changes in the lower gut; DPP-4 inhibitors, prolonging sotagliflozin-induced GLP-1 secretion) give hope for significant positive interactions. But again, specific studies are needed.

In conclusion, sotagliflozin seems to represent a promising treatment for both type 1 and type 2 diabetes, either alone or in combination with metformin or DPP-4 inhibitors in type 2 diabetes or, with an adequate insulin protocol, in type 1 diabetes. The dual inhibition of both SGLT-1 and SGLT-2 improves the efficacy of this SGLT inhibitor also in mild and severe CKD, suggesting an extended use also in frail patients where therapeutic options are currently limited.

## Abbreviations

SGLT2: sodium–glucose co-transporter-2
SGLT1: sodium–glucose co-transporter-1
GLUT1: glucose transporter-1
GLUT4: glucose transporter-2
T2D: type 2 diabetes
T1D: type 1 diabetes
SGLTis: sodium–glucose co-transporter inhibitors
MACE: Major Adverse Cardiovascular Events
PPG: post prandial glucose
FPG: fasting plasma glucose
UGE: urinary glucose excretion
GI: gastro-intestinal
GLP-1: glucagon like peptide-1
GIP: gastro inhibitor peptide
PYY: intestinal peptide YY
DPP-IV: di-peptidil-peptidase IV
SCFAs: short chain fatty acids
FFA: free fatty acids
SH: severe hypoglycemia
DKA: diabetic ketoacidosis
